# β-Thalassemia Major Complicated by Acute Myeloid Leukemia

**DOI:** 10.7759/cureus.69557

**Published:** 2024-09-16

**Authors:** Mehreen Khalid, Maymoona Suhail, Alizah Faisal, FNU Poombal

**Affiliations:** 1 Department of Hematopathology, Armed Forces Institute of Pathology, Rawalpindi, PAK; 2 Department of Hematology, Armed Forces Institute of Pathology, Rawalpindi, PAK; 3 Department of Hematology, Rawalpindi Medical University, Rawalpindi, PAK; 4 Department of Pathology, Nishtar Medical University, Multan, PAK

**Keywords:** acute myeloid leukemia (aml), hematological malignancies, iron overload, transfusion-dependent anemia, β-thalassemia major

## Abstract

This report describes the rare co-occurrence of acute myeloid leukemia (AML) French-American-British type M2 in a 4.5-year-old boy with previously diagnosed thalassemia major, an inherited hemoglobinopathy, typically presenting with severe, transfusion-dependent anemia. Chronic transfusions, though lifesaving, can lead to iron overload, which may generate free radicals and potentially contribute to malignancy. Our case highlights the importance of close monitoring for secondary malignancies in thalassemia patients. Our patient born to consanguineous parents, presented with persistent fever, abdominal pain, and splenomegaly. Hematological investigations revealed severe cytopenias (low blood cell counts) and many immature blood cells (blasts). Bone marrow examination confirmed AML M2, characterized by an overabundance of myeloid blasts. Despite the initiation of myeloid leukemia-directed aggressive chemotherapy, the patient, unfortunately, succumbed to the disease within a month of diagnosis.

## Introduction

β-Thalassemia is a group of heterogeneous inherited disorders caused by the reduced or absent synthesis of the β-globin chain and consequent accumulation of α-globin chains in red blood cells (RBCs). β-Thalassemias are autosomal recessive disorders, with β-thalassemia major being the most severe form. Transfusion-dependent anemia is the hallmark of β-thalassemia. Untreated or poorly transfused patients may exhibit symptoms such as stunted growth, pallor, jaundice, and skeletal changes [1]. The condition requires lifelong support and medical care, with stem cell transplantation being the only definitive cure. International guidelines recommend a multidisciplinary approach to managing thalassemia [2]. Genetic analysis has identified nearly 200 point mutations and rare deletions that cause β-thalassemia [3].

Patients with β-thalassemia major are increasingly reported to be more susceptible to developing malignancies, especially at a younger age, compared to the general population. This increased risk necessitates the implementation of cancer screening protocols for these patients [4]. The hallmark of β-thalassemia major is transfusion-dependent anemia, which often leads to iron overload due to frequent blood transfusions. The excess iron is deposited in various organs, including the liver, heart, and pancreas. Iron accumulation can produce toxic oxygen-free radicals, which modify the immune system and potentially lead to the development of malignancies [5].

## Case presentation

History of presentation

A four-and-a-half-year-old male, a product of consanguineous marriage, otherwise developmentally normal and fully immunized, was diagnosed with thalassemia major at six months of age through hemoglobin electrophoresis. The patient required blood transfusions every six months until the age of two years, with the frequency of transfusions increasing to weekly by the age of four. It was noted that the patient had not undergone any investigations to rule out iron overload, nor was he initiated into any iron chelation therapy.

Physical examination

The patient presented with persistent fever for two weeks, ranging from 99°F to 101°F, associated with abdominal pain, distention, cough, decreased appetite, and worsening fatigue. On examination, the child was vitally stable but appeared extremely pale, lethargic, and irritable, with a protuberant abdomen. He had marked bruising on the anterior aspects of both legs, measuring 2 × 4 cm and non-blanching. The patient has massive splenomegaly reaching the right iliac fossa, with no signs of hepatomegaly. The rest of the systemic examination was unremarkable.

Laboratory findings

**Figure 1:**
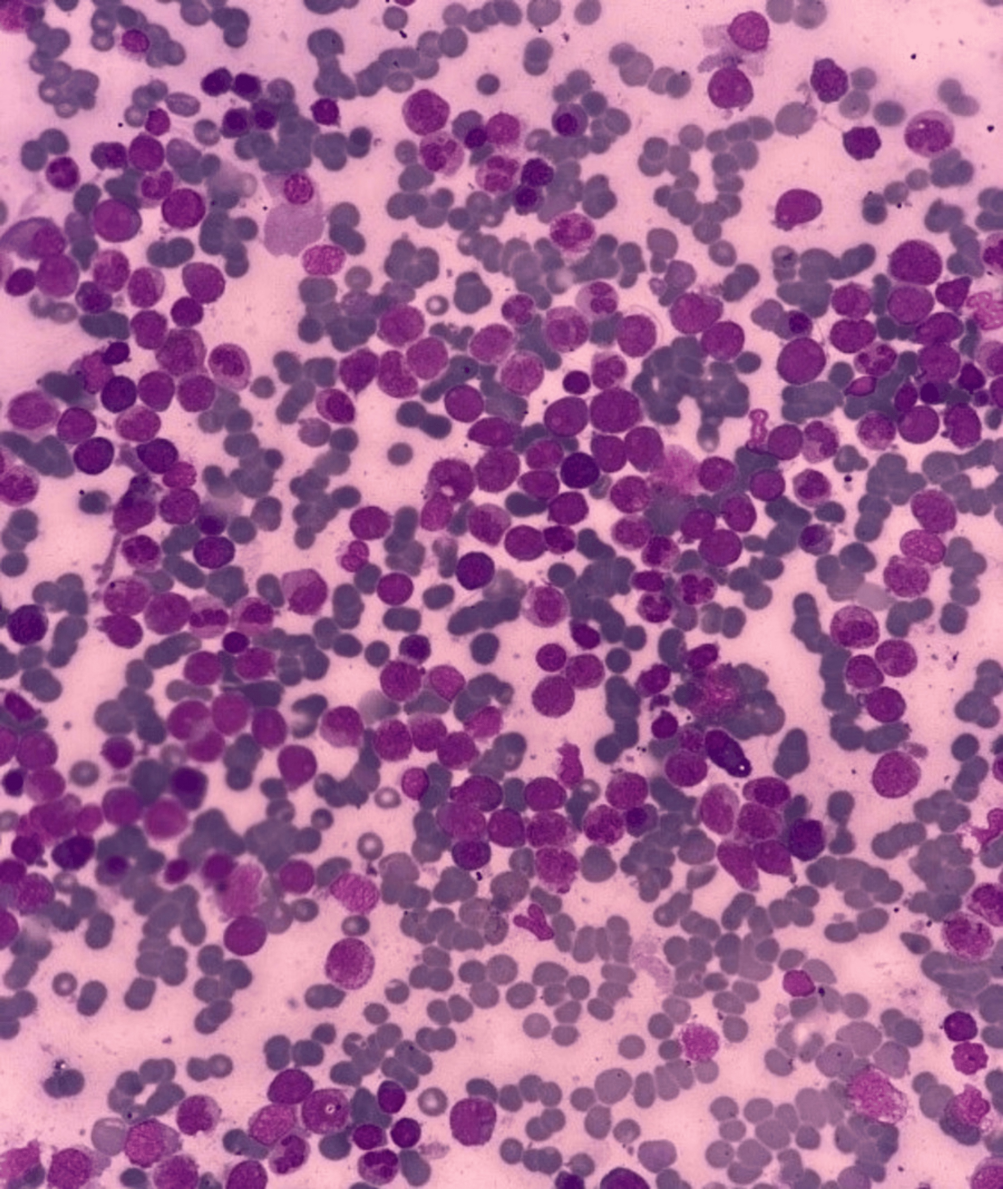
Leishman-stained peripheral blood smear at 40× magnification, showing a high percentage of blasts with prominent nucleoli and low nucleus-to-cytoplasm ratio, indicative of acute myeloid leukemia

Laboratory findings are presented in Table 1. Red cell morphology indicated hypochromia, microcytosis, and occasional fragmentation. Morphologically, the blasts were medium to large in size with a low nucleus-to-cytoplasm ratio, dispersed nuclear chromatin, and prominent nucleoli, indicating acute myeloid leukemia (AML), as shown in Figure 1.

**Table 1:** Laboratory findings RBCs: red blood cells; WBCs: white blood cells

Parameters	Patients values	Reference values
Complete blood count		
Hemoglobin (g/dL)	5.3	12-15
Platelet count (×10^9^/L)	16	150-450
Total leukocyte count (×10^9^/L)	33	4-10
Differential count		
Neutrophils (%)	7	40-80
Lymphocytes (%)	37	20-40
Monocytes (%)	2	2-10
Eosinophils (%)	0	1-6
Myelocytes (%)	3	0
Blast cells (%)	51	0
Nucleated RBCs	130 nucleated RBCs/100 WBCs	0
Serum iron profile		
Serum ferritin (ng/mL)	>4,000	30-350

Bone marrow evaluation

**Figure 2:**
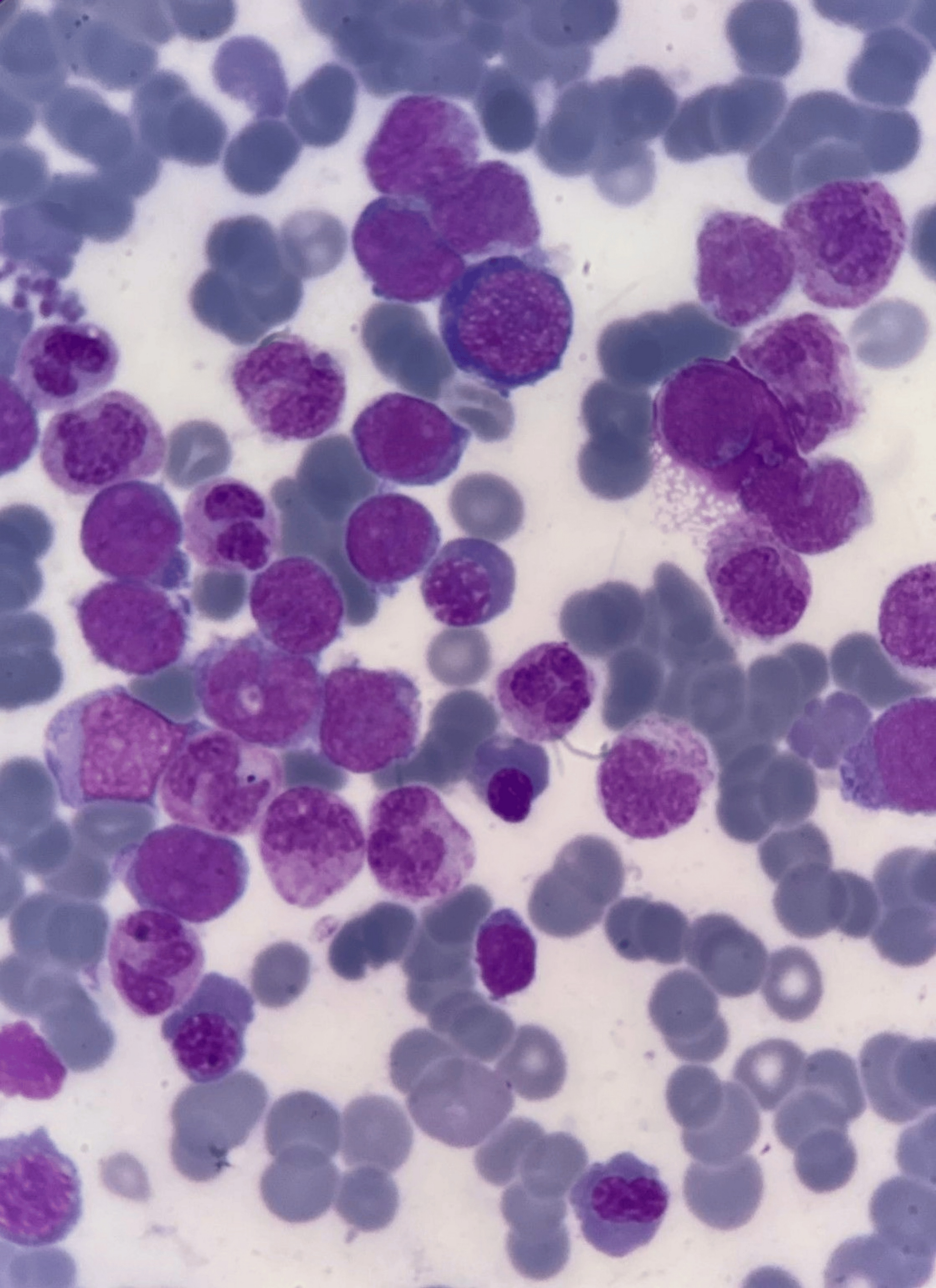
Leishman-stained bone marrow aspirate at 100× magnification, demonstrating a predominance of blast cells with conspicuous nucleoli and low nucleus-to-cytoplasm ratios, typical in acute myeloid leukemia FAB M2 FAB: French-American-British

**Figure 3:**
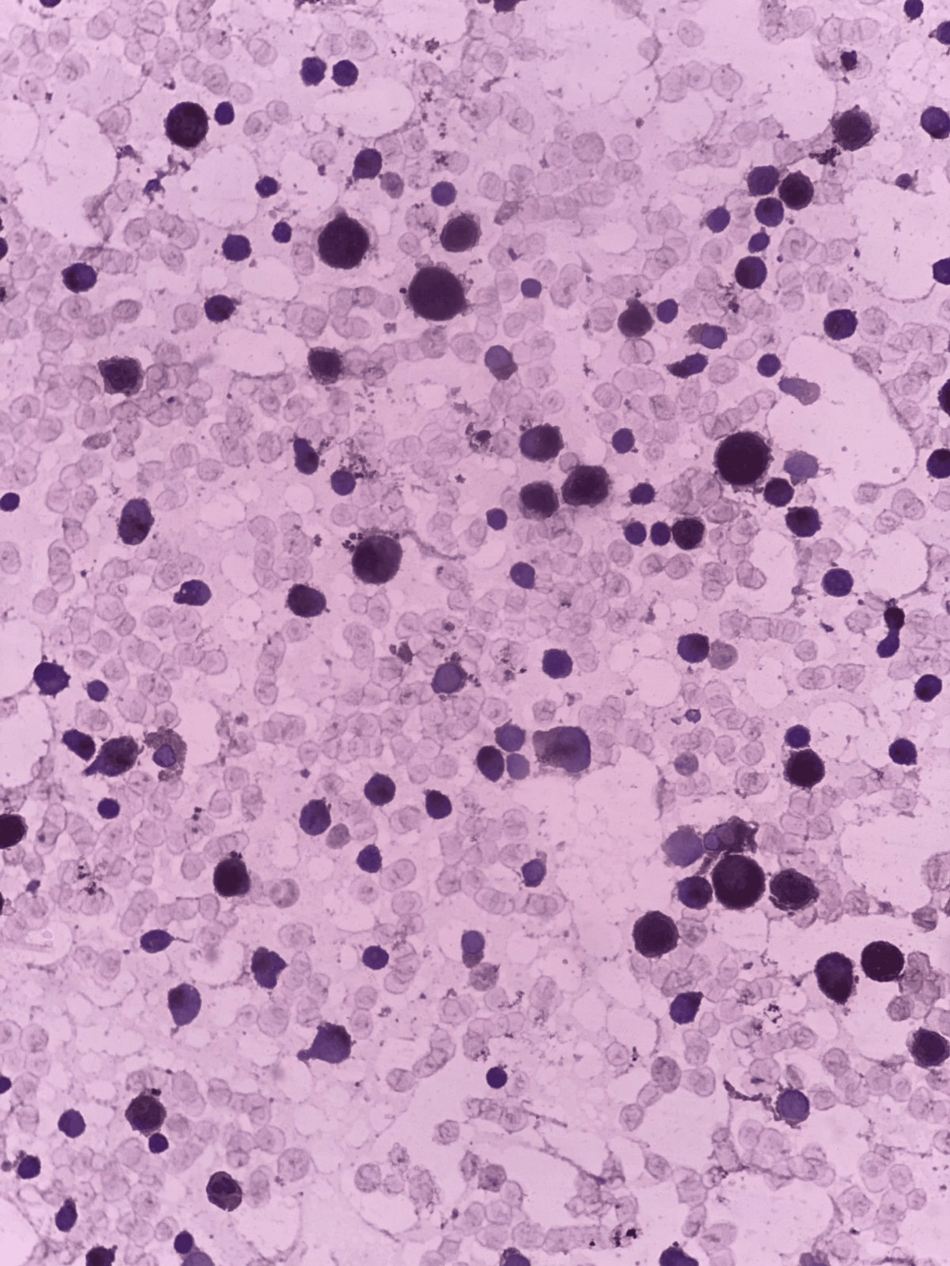
Sudan black B staining at 40× magnification showing positive myeloid blast staining, which confirms the myeloid lineage of the cells, consistent with acute myeloid leukemia FAB M2 FAB: French-American-British

Given the patient's massive splenomegaly, cytopenias, high blast percentage, and increased nucleated RBC count, bone marrow aspiration and trephine biopsy were advised. Bone marrow aspiration showed hypercellular fragments and trails, with hyperplastic, normoblastic erythropoiesis exhibiting megaloblastic and dyserythropoietic changes. Myelopoiesis was depressed, and megakaryocytes were absent. The blast percentage was 22%, with medium- to large-sized blasts showing prominent nucleoli and a low nucleus-to-cytoplasm ratio (Figure 2). Sudan black B stain was positive in blast cells (Figure 3).

The morphological appearance of blasts and Sudan black B stain confirmed myeloid origin. The trephine biopsy findings were consistent with the aspiration report. Hyperplastic erythropoiesis was attributed to thalassemia major. The high blast count in peripheral blood and bone marrow, positive for Sudan black B, suggested a provisional diagnosis of AML. Immunophenotyping was performed to confirm the diagnosis.

On immunophenotyping (flow cytometry), 30% of the gated population was positive for weak CD45, CD34, CD117, and human leukocyte antigen DR. The blast also expressed myeloid lineage markers CD13 and CD33. Other lineage-specific markers were negative in the blast population.

Diagnosis

Based on the peripheral smear, bone marrow aspiration biopsy, and immunophenotyping, a diagnosis of AML French-American-British type M2 was confirmed. Polymerase chain reaction for AML gene markers was negative, and cerebrospinal fluid routine examination was normal.

Treatment

The child was treated for AML M2. His marrow had less than 2% blasts by the eighth day postchemotherapy. Although he initially responded well to chemotherapy, he could not tolerate the treatment and died within one month of starting it.

## Discussion

β-thalassemia is a genetic disorder caused by defects in hemoglobin synthesis due to decreased production of β-globin chains. This defect leads to the accumulation of α-globin chains in RBCs [3,6]. β-thalassemia is endemic in tropical and subtropical areas, particularly in the Indian subcontinent and the Mediterranean region. It is estimated that 5%-7% of the world's population carries a mutated gene affecting the production or function of hemoglobin [7].

Patients with β-thalassemia typically present with anemia due to hemolysis and ineffective erythropoiesis [8]. The bone marrow of these patients shows hyperplasia of erythroid precursors, increased apoptosis of erythroblasts during their final differentiation stage, and an increased number of macrophages. Causes of ineffective erythropoiesis include oxidative stress from excess α-globin chain due to α/β-globin chain imbalance, iron overload, and various hormonal, cytokine, and environmental factors [9]. Increased apoptosis results in the extracellular expression of phosphatidylserine on dying erythroid precursors, signaling their removal by macrophages [10].

Treatment for β-thalassemia includes regular red cell concentrate transfusions, precise iron chelation therapy, inducers of fetal hemoglobin, and supportive care. A definitive treatment option is allogeneic stem cell transplantation. These treatments have significantly increased life expectancy and improved the quality of life for patients with β-thalassemia [11]. However, regular blood product transfusions lead to iron deposition in various organs. Excess-free iron, rapidly taken up by transferrin, becomes saturated, resulting in toxic non-transferrin-bound iron (NTBI). NTBI generates reactive oxygen species through the Fenton reaction, causing oxidative stress [12]. Platelets and erythrocytes in these patients exhibit increased reactive oxygen species and decreased intracellular glutathione [13].

Hepcidin, the regulator of iron status, is found at low levels in these patients compared to high free iron levels [14]. Serum ferritin levels, although indicative of body iron status, are unreliable in the presence of liver disease. However, these patients' serum iron and transferrin saturation are more reliable [15]. With increased survival rates, new complications like malignancies have emerged [16].

The occurrence of hematological malignancies in β-thalassemia patients remains a mystery, potentially due to genetic and environmental factors. Quattrin et al. found no genetic association between lymphoma and thalassemia, with comparable leukemia incidence in the general population and β-thalassemia patients [17]. A retrospective study (1999-2020) identified the leading causes of death in thalassemia patients as cardiovascular (26.0%), hematological (20.7%), malignancy (17.8%), endocrine (6.3%), respiratory (6.2%), nervous system disorders (5.0%), gastrointestinal (GI; 4.9%), psychiatric (3.3%), infection (3.0%), and other causes (6.7%) [18].

One plausible theory links the toxic effects of iron overload to malignancy development. Studies have shown that elevated iron levels promote tumor growth, with high iron concentrations found in macrophage deposits within tumors [19]. This theory is particularly relevant to β-thalassemia patients who suffer from transfusion-dependent anemia. Repeated transfusions can cause immunomodulation and excessive iron deposition. NTBI generates free oxygen radicals and reduces intracellular glutathione [13]. Decreased hepcidin levels exacerbate iron absorption from the GI tract and release from macrophages, complicating the condition further. The potential for familial cancer syndromes, combined with acquired factors like transfusion-induced immunomodulation, cannot be overlooked.

We emphasize the critical need for regular cancer screenings for thalassemia patients and further research to elucidate the precise mechanisms linking iron overload to the development of malignancies. A multidisciplinary approach, incorporating close monitoring and early intervention strategies to mitigate the risk of secondary complications like AML, is crucial. By implementing these measures, we can improve outcomes for thalassemia patients by facilitating the early detection and treatment of co-occurring malignancies.

## Conclusions

β-thalassemia is a genetic disorder that affects multiple organ systems and presents with significant complications. Although treatment advancements have improved patient outcomes, they pose new challenges, such as the risk of iron overload and its potential connection to malignancies. While the exact link between iron overload and cancer is not fully understood, it is crucial to prioritize ongoing research, regular cancer screenings, and a multidisciplinary approach to patient care. Focusing on early detection and intervention can enhance the management of β-thalassemia, improving both quality of life and reducing the risk of serious complications.
